# Prospective analysis of salivary melatonin levels in patients with symptomatic pineal cysts

**DOI:** 10.1007/s00701-026-06851-1

**Published:** 2026-04-14

**Authors:** Xenia Hautmann, Fares Komboz, Tammam Abboud, Dorothee Mielke, Veit Rohde

**Affiliations:** 1 Department of Neurosurgery, University Hospital Göttingen, Göttingen, Lower Saxony Germany; 2 Department of Neurosurgery, University Hospital Augsburg, Stenglinstr. 2, 86156 Augsburg, Germany

**Keywords:** Pineal cyst, Melatonin, Cephalgia, Sleep disorder

## Abstract

**Background:**

Melatonin (ML) is synthesized in the pineal gland and is subject to a circadian rhythm. It significantly influences the sleep–wake cycle. Patients with non-hydrocephalic symptomatic pineal cysts (PC) suffer from a variety of symptoms including cephalgias and sleep disorders. However, the underlying pathomechanism remains unclear. Among others, a disturbed ML secretion due to the cyst is being discussed. However, the data regarding ML levels in patients with symptomatic PC is still insufficient.

**Methods:**

As part of a prospective study in non-hydrocephalic patients with symptomatic PC, we examined the preoperative ML level in the saliva in a half-hour rhythm between 08:30 p.m. and 01:00 a.m.. In addition, symptoms, in particular sleep quality and headache, were recorded over a week using a diary. We analyzed the relationship between symptoms and the ML levels including their time course during early night hours.

**Results:**

A total of ten female patients were included. The average diameter of the PC was 11 mm. Patients were able to sleep through the night on an average of 2.5 out of 7 days (range 0 – 6 days), problems falling asleep occurred on an average of 3.6 out of 7 days (range 0 – 7 days). Headaches occurred on an average of 5.5 out of 7 days (range 2 – 7 days). The severity of the headaches ranged from 3 to 7 on the visual analog scale (VAS). Four patients (40%) reported nausea. There was no significant correlation between ML levels and the recorded symptoms. However, the ML values showed a lack of increase during the night. In six patients, ML levels were within the normal range at 8:30 p.m. (*p* = 0.001). After 10:00 p.m. only two patients (*p* < 0.0001), and after 00:30 a.m. all patients had pathologic ML levels (*p* < 0.0001). Of these patients, two had ML values above, and seven below the normal range.

**Conclusions:**

The ML levels in patients with symptomatic PC show a deficient nocturnal increase, which may provide an explanation for the symptoms.

**Supplementary Information:**

The online version contains supplementary material available at 10.1007/s00701-026-06851-1.

## Introduction

Pineal cysts (PC) are benign, often incidentally discovered lesions of the pineal gland with a reported prevalence of up to 37.5% in MRI studies [24]. Many PCs remain asymptomatic, but PC patients also complain about headache, sleep disturbances, dizziness, and nausea [1, 5, 7]. Patients with PCs are about twice as likely to suffer from headaches compared to patients in a control group without PC [18]. The pathophysiological relationship between these symptoms and the PC has not yet been conclusively clarified. In some cases, PC can cause hydrocephalus or Parinaud syndrome; here, surgical treatment is the gold standard. However, the available data show that even patients with symptomatic PC who do not suffer from hydrocephalus benefit from cyst resection and cyst fenestration as well [6, 11]. In addition to mechanical explanations such as compression of the internal cerebral veins or intermittend obstruction of the aqueduct [2], a disturbed melatonin (ML) release is also being discussed. In animal experiments, there were no differences in the sleep–wake cycle before and after pinealectomy [8]. Slawik et al. conducted a prospective study on patients with pineal tumors, in which ML production in saliva was examined preoperatively and postoperatively. Postoperatively, a significant decrease in ML was found in the nighttime saliva of all patients with no significant effect on the patients' sleep–wake cycle [22]. Concerning non-hydrocephalic patients with symptomatic PC, data of ML levels is lacking.

ML (N-acetyl-5-methoxytryptamine) is synthesized in the pineal gland, which is a central neuroendocrine organ that plays a key role in regulating the circadian rhythm. The production of this hormone is subject to a circadian rhythm, with secretion typically peaking during the dark phase of the night. Pineal ML secretion is regulated by the hypothalamic nuclei and by the action of light via a multisynaptic pathway starting from retinal ganglion cells [12, 14]. Although ML can also be produced in other tissues, the systemically measurable proportion in saliva and blood mainly originates from the pineal gland [4]. However, human ML production is subject to interindividual variability. The greatest increase in ML concentration in serum has been observed in previous studies between 8 p.m. and 2 a.m. [3]. The maximum ML concentration in serum was reached between 2:00 a.m. and 6:00 a.m., and in urine between 6:00 a.m. and 8:00 a.m. [3]. ML peak is reached slightly earlier in saliva than in blood samples (between midnight and 3 a.m.) [16]. Previous studies have shown the highest measurement accuracy when measuring ML in serum every 20–30 minutes [3]. However, this requires a venous catheter, which is why we have chosen to use saliva samples for this study as part of the risk–benefit assessment. Lewy already described in 1983 that this type of ML measurement is least prone to error when performed in dim light [13]. Previous studies on ML measurement in saliva were conducted with a sampling interval of 30 min [3], with the authors recommending frequent measurements during the period when the greatest change is expected. Intraindividual ML measurements are considered stable [3] while interindividual differences make the establishment of reference values difficult.

The aim of this study is to quantify the preoperative ML levels of patients with symptomatic PC and to correlate them with symptoms.

## Methods

We conducted a monocentric prospective study on non-hydrocephalic patients with symptomatic PC between March 2023 and May 2024. Ethical approval was obtained by the ethics committee of the University Hospital Göttingen (No. 16/1/23).

### Patients

We included patients who were over 18 years of age with evidence of a symptomatic PC on MRI scan without evidence of hydrocephalus or a pineal tumor. We defined typical symptoms as headaches, difficulty falling asleep and staying asleep, and visual disturbances, after other underlying pathologies had been ruled out by MRI, neurological and, where necessary, ophthalmological examinations. Previous illnesses and current medication were recorded with a particular focus on migraine, hormonal disorders, and any prior neurosurgical procedures. ML intake during the last 2 weeks was an exclusion criterion. In addition, the duration of symptoms in years and the year of initial diagnosis were recorded.

### MRI

**Fig. 1:**
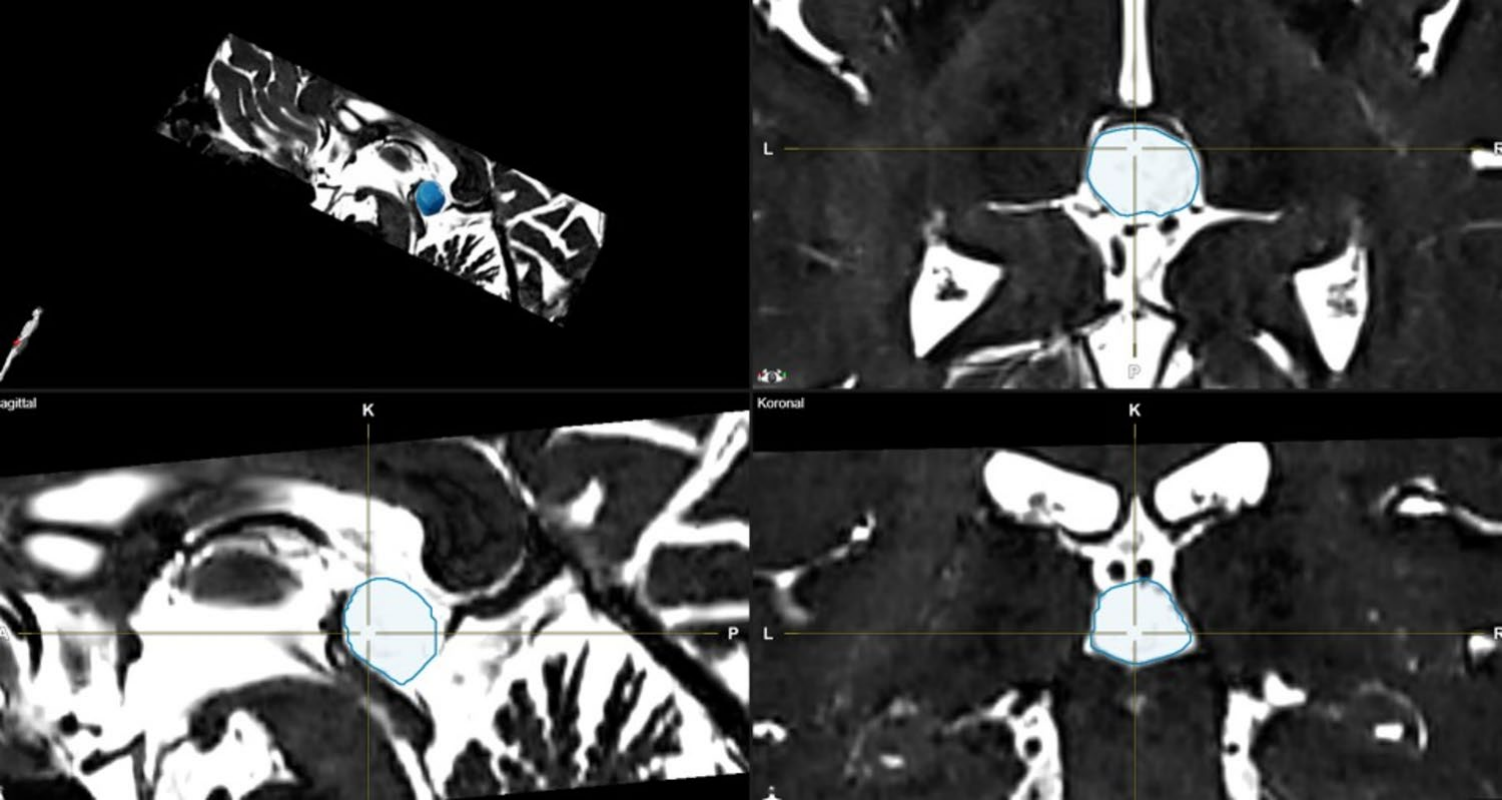
Volumetric measurement of PC in T2

The MRI scan had to be no more than two years old, without new symptoms since the MRI scan. The anteroposterior (ap) diameter of the PC was measured in the sagittal T2-weighted images. Using these T2-weighted MRI scans, the volume of the PC was calculated using Brainlab software (Brainlab, Munich, Germany) (Fig. 1).

### Assessment of specific symptoms

Headache and sleep disturbances were assessed over the course of a week at home in the form of a diary. Difficulties falling asleep, difficulties staying asleep, and the duration of sleep per day were recorded, as well as the frequency and severity of headaches using the Visual Analog Scale (VAS). In addition, visual disturbances and nausea were queried, and specific or nonspecific symptoms were recorded using free text.

### Melatonin level measurement

Salivary ML levels were measured preoperatively on the first night following hospital admission (two days before scheduled surgery), with samples collected between 8:30 p.m. and 1:00 a.m.. To reduce physical and psychological stress, we decided against later measurement times. In order to rule out short-term fluctuations and measurement errors, measurements were taken every 30 minutes, even if reference values were not available for each specific point in time. Patients were instructed not to brush their teeth, eat or drink two hours before and during the measurement period. 1–2 hours before the measurement, the room was darkened and compliance with dim light exposure was randomly checked by the nursing staff. Patients were not allowed to use bedside lamps and were restricted in their use of cell phones; watching TV or reading was not permitted. Patients were instructed to lie down in bed and rest as if they were trying to fall asleep.

The lighting conditions only allowed them to orient themselves in the room in order to provide the samples. The saliva samples were collected using a straw without any stimulating measures. After receiving appropriate instructions, the patients independently provided saliva samples in a test tube for each measurement, and the samples were then stored in a dark box. The patient room was kept dimly lit throughout the measurement period. Sample collection was regularly supervised by a nurse. ML levels were determined in an external laboratory. The samples were sent in a dark container the morning after the measurement. ML levels were then determined using Enzyme-Linked Immunosorbent Assay (IBL).

### Statistics

Statistical analysis was performed using SPSS (IBM, Version 30.0). We analyzed the relationship between symptoms and the ML level using Spearman's correlation and Mann–Whitney-U test. To assess whether the measured salivary ML concentrations in the subjects significantly deviate from expected reference values, two statistical tests were applied. First, a binomial test was performed to evaluate whether the proportion of measurements falling within the normative reference range (assumed 95%) differs significantly from this expected proportion. The null hypothesis for this test states that the success probability (measurements within the reference range) equals 0.95. Second, to analyze the continuous concentration values, a Wilcoxon signed-rank test was conducted to determine if the median difference between the observed values and the reference values differs significantly from zero, without assuming normal data distribution. All tests used a significance level of 0.05.

## Results

**Fig. 2:**
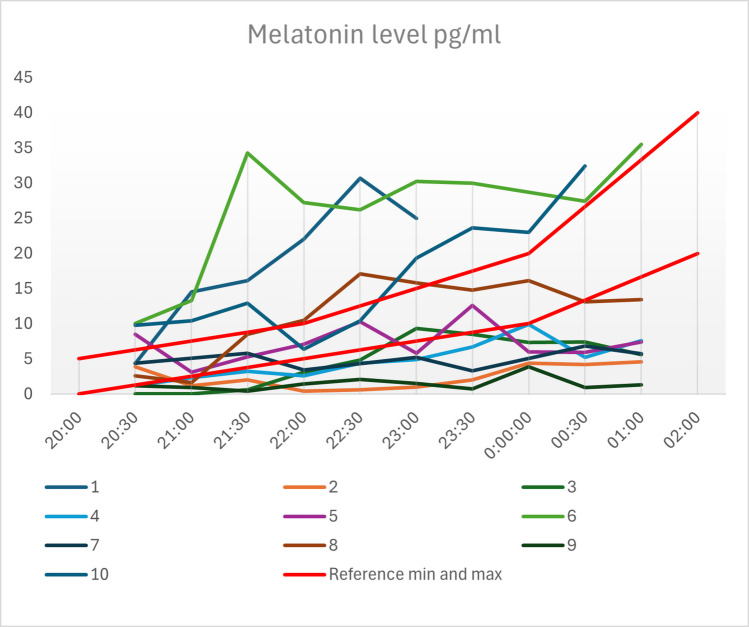
ML levels per patient throughout the measuring period. The progression of melatonin concentration per individual is shown in the respective colors. The red lines delimit the reference values

Between March 2023 and May 2024, 10 patients were prospectively included. All patients were female. The average age was 37.8 years (range 24–45 years). The average ap diameter of the PC was 11 mm (range 4 −18 mm), mean volume was 1.7 cm^3^ (range 0.4–4.2 cm^3^). Due to incompatibility between the external MRIs and Brainlab software, the volume could only be determined for six patients. The patients had been suffering from symptoms for an average of 4.5 years (range 0.5–14 years). Six patients reported problems falling asleep and seven reported problems staying asleep. All patients reported headaches that affected their daily lives. They were able to sleep through the night on an average of 2.5 out of 7 days (range 0–5 days), problems falling asleep occurred on an average of 3.6 out of 7 days (range 0–7 days). Headaches occurred on an average of 5.5 out of 7 days (range 2–7 days). Four of the ten patients suffered from continuous headaches. The severity of headaches ranged from 3 to 7 on the VAS. Four patients (40%) reported nausea. Visual disturbances and fatigue were only present in two patients (20%). There was no significant correlation between the size (volume or diameter) of the PC and the ML level and between low ML levels and the symptoms recorded (sleep disturbance *p* = 0.866, sleep duration *p* = 0.381, difficulty falling asleep *p* = 1.00, frequency of headaches *p* = 1.00, days with persistent headaches *p* = 0.857, intensity of headaches *p* = 1.00, nausea *p* = 0.406, fatigue *p* = 1.00, visual disturbances *p* = 1.00). A longer time since onset of symptoms correlated significantly with a decreased ML concentration at 10:30 p.m. (*p* = 0.031). However, according to binominal testing, the ML values significantly deviated from the reference values, particularly due to a lack of increase during the night. Figure 2 shows the time course of ML concentration per patient during the measurement period. At 8:30 p.m., six patients had a ML level within the normal range (*p* = 0.001). After 10:00 p.m., only two patients (*p* < 0.0001) had a normal ML level. After 00:30 a.m. all patients had ML levels outside the normal range (*p* < 0.0001), with ML levels above the normal range in two patients, and below the normal range in eight patients. The deviation from the mean reference value was statistically significant for the measurements at 8:30 p.m. (*p* = 0.039) and 01:00 a.m. (*p* = 0.023). Two patients did not complete the measurement due to falling asleep. The measurement at midnight for patient 6 was above 400 pg/ml and was interpreted as an erroneous measurement and removed from the evaluation.

## Discussion

This prospective study is one of the first to examine ML levels in non-hydrocephalic patients with symptomatic PC. To date, mainly three underlying pathologies have been discussed as possible causes of the typical symptoms experienced by patients with PC. Those are compression of the internal cerebral veins, intermittent obstruction of the aqueduct and a change in ML levels.

The main finding of this study is a noticeable lack of increase of ML during the night in most patients. While six patients were still within the normal range at 8:30 p.m. (*p* = 0.001), the number significantly dropped to two patients at 10:00 p.m. and to zero at 00:30 a.m., with 2 patients being above and seven patients being below the normal range. However, due to interindividual differences and limited data, the reference values must be interpreted with caution. The deviation at individual measurement points can only be assessed to a limited extent. However, the lack of an increase during the night is relevant. In other studies, the determination of ML in saliva in healthy subjects showed an increase in its course corresponding to that of blood samples. This cannot be observed in our cohort of PC patients. Benloucif et al. showed ML levels of approximately 5 pg/ml at 8 p.m. and approximately 20 pg/ml at 10 p.m. Analysis of individual patient factors, such as symptoms and cyst size, did not reveal any significant differences between the patients. This finding requires further investigation in a larger cohort.

In 2013, Májovsky et al. investigated serum ML levels in five symptomatic and two asymptomatic patients with PC [15]. However, sleep disorders were not recorded, but mainly headaches and dizziness as well as neurological focal deficits. No significant difference was found between symptomatic and asymptomatic patients, although ML levels were slightly lower in symptomatic patients. They therefore propagated normal ML secretion in patients with PC. However, that study did not look at any deviation from reference values due to possible individual variations. Our study with symptomatic patients, however, revealed a different situation. We observed noticeably low ML values, especially after 10 p.m. There were even significant deviations for the times 8:30 p.m. and 01:00 a.m. Another difference between the studies is the measurement method using blood samples or saliva. For our study, patients had to be woken up every 30 min to provide saliva samples. For a hormone that is directly related to the sleep cycle, this must be discussed as a possible limitation. On the other hand, several studies have rated the measurement of ML in saliva as reliable [3, 17, 23]. Despite waking the test subjects, similar peaks were observed in healthy subjects as in blood samples, although the absolute values in saliva are lower. In our cohort, there was no statistically significant correlation between the documented symptoms and the deviation of ML levels below the norm. Since all patients were symptomatic, the variance in the cohort was probably too small to achieve significance.

Due to the small number of cases and the lack of a healthy comparison cohort, there are limitations regarding the statistical significance in our cohort. Nevertheless, our results show a very noticeable tendency toward a lack of increase in ML at night in non-hydrocephalic patients with symptomatic PC. Furthermore, we consider our results to be valid, as previous studies on the reproducibility of ML measurements showed no relevant intraindividual variation over a period of several weeks [19]. The fact that our cohort consists solely of women does not mitigate this through possible hormonal influences, since previous studies showed no fluctuation in ML concentration depending on the menstrual cycle [20]. Moreover, we did not include patients who had taken ML within the last two weeks, in order to allow sufficient time between the last oral ML substitution and both the period during which symptoms were recorded and the period during which ML was measured in saliva. Studies have shown an almost complete degradation of the substituted ML after 4 h, so we are definitely outside this time frame [21]. Nevertheless, depending on the study and measurement method, the reference values given vary slightly, but are always above the references we assume and thus above those of our patients with PC [16, 23]. While Májovsky et al. measured serum ML, we decided to measure saliva ML, since it is less invasive. Although the concentration of ML in saliva is lower than in serum, it is reproducibly subject to the same circadian rhythm [16, 23]. Therefore, we consider our results to be sufficiently valid to conclude that ML levels in patients with symptomatic PC are below normal.

It is also important to note that we focused on sleep disturbances among the symptoms, which were not explicitly mentioned in Májovsky's study. We therefore consider our cohort to be particularly significant, as ML plays a key role in the sleep cycle, even in healthy individuals, and because sleep disturbances are present in up to 21—27% of patients with symptomatic PC [9, 11].

### Limitations

Although our prospective study provides important insights into ML levels in patients with symptomatic PC, it has limitations. The sample size, and consequently the statistical power, is low. Furthermore, no control group with asymptomatic PC was analyzed. Due to interindividual variations, the determination of ML reference values and thus the interpretation of deviations of individual values from these can only be assessed to a limited extent. The external MRI scans did not allow the use of sequences required to analyze thalamic edema as a differential diagnosis due to potential venous compression. On the other hand, other studies have shown that ADC is not an adequate diagnostic tool in patients with PC, and there was no evidence of thalamic edema as the cause [10]. As a next step, a multicenter study should investigate both symptomatic and asymptomatic PC patients, and the influence on postoperative outcome needs to be clarified. Nonetheless, we consider our results a significant contribution to PC research and an important basis for future studies. This includes investigation in a larger cohort, postoperative measurement of ML, and analysis of oral ML substitution as a treatment option.

## Conclusion

The ML level in non-hydrocephalic patients with symptomatic PC shows a lack of increase at night, which may provide an explanation for the symptoms. As a next step more symptomatic and asymptomatic patients need to be analyzed, as well as the influence of ML levels on the postoperative outcome.

## Supplementary Information

Below is the link to the electronic supplementary material.

ESM 1 Supplementary Material 1 (DOCX 16.5 KB)

## Data Availability

The data can be requested from the corresponding author if required.

## Declarations

### Previous presentation of data

The results of this study were presented in advance at the Annual Meeting of the German Society for Neurosurgery in Hannover, June 2025 and at the Annual Meeting of the European Association of Neurosurgical Societies in Sofia, October 2024.
