# Supplementary material for: Prospective analysis of salivary melatonin levels in patients with symptomatic pineal cysts
Source: Acta Neurochir (Wien). 2026 Apr 14;168(1):97. doi: 10.1007/s00701-026-06851-1 (PMC13083344; doi:10.1007/s00701-026-06851-1)
Supplement: Supplementary file 1 — Supplementary Material 1 (DOCX 16.5 KB) [file 701_2026_6851_MOESM1_ESM.docx]

Pseudonym Patient:

Zusammenhang zwischen dem präoperativen Melatoninspiegel und Symptomen bei Pinealiszysten

**Kopfschmerz- und Schlaftagebuch in der Woche vor der Operation**

|  | **Tag 1** | **Tag 2** | **Tag 3** | **Tag 4** | **Tag 5** | **Tag 6** | **Tag 7** |
| --- | --- | --- | --- | --- | --- | --- | --- |
| **Datum** |  |  |  |  |  |  |  |
| **Durchgeschlafen?** |  |  |  |  |  |  |  |
| **Wie viele Stunden haben Sie geschlafen?** |  |  |  |  |  |  |  |
| **Einschlafprobleme?** |  |  |  |  |  |  |  |
| **Kopfschmerzen gehabt?** |  |  |  |  |  |  |  |
| **Wie häufig an diesem Tag hatten Sie Kopfschmerzen?** |  |  |  |  |  |  |  |
| **Wie stark waren die Kopfschmerzen auf einer Skala von 0-10?** (0= keine, 10= sehr starke Schmerzen) |  |  |  |  |  |  |  |
| **Andere Beschwerden/ Besonderheiten?** |  |  |  |  |  |  |  |
